# Prognostic role of E2F1 gene expression in human cancer: a meta-analysis

**DOI:** 10.1186/s12885-023-10865-8

**Published:** 2023-06-05

**Authors:** Jingjing Li, Wen Bi, Fang Lu, Bei Pan, Mengqiu Xiong, Lubanga Nasifu, Zhenlin Nie, Bangshun He

**Affiliations:** 1 Department of Laboratory Medicine, Nanjing First Hospital, Nanjing Medical University, Nanjing, 210006 China; 2 School of Basic Medicine and Clinical Pharmacy, China Pharmaceutical University, Nanjing, China; 3 Department of Pharmacy, Nanjing First Hospital, China Pharmaceutical University, Nanjing, China; 4 Medical College, Southeast University, Nanjing, 210006 China; 5 Department of Biology, Muni University, Arua, Uganda; 6 Collaborative Innovation Center for Cancer Personalized Medicine, Nanjing Medical University, Nanjing, 210006 China

**Keywords:** E2F1, Cancer, Breast cancer, Prognosis, Meta-analysis, Gene expression, Overall survival, Disease-free survival

## Abstract

**Objective:**

E2F1 has been confirmed to be highly expressed in a variety of cancers. To better understand the prognostic value of E2F1 in cancer patients, this study was conducted to comprehensively evaluate the prognostic value of E2F1 in cancer according to published data.

**Method:**

PubMed, Web of Science and CNKI database were searched until May 31^th^, 2022 by using key words to retrieve the published essays on the role of E2F1 expression in the prognostic value of cancer. The essays were identified according to the inclusion and exclusion criteria. The pooled result of hazard ratio and 95% confidence interval was calculated with Stata17.0 software.

**Result:**

A total of 17 articles were included in this study involved in 4481 cancer patients. The pooled results showed that higher E2F1 expression was significantly correlated with unfavorable overall survival (HR = 1.10, *I*^2^ = 95.3%, *P_Heterogeneity_ = 0.000) and disease-free survival (HR = 1.41,* I*^2^ = 95.2%, *P_Heterogeneity_ = 0.000) of cancer patients. Such a significant association of was maintained subgroup of sample size of patients (> 150: for OS, HR = 1.77, and for DFS, HR = 0.91; or < 150: for OS, HR = 1.93, and for DFS, HR = 4.39), ethnicity (Asian: for OS, HR = 1.65, and for DFS, HR = 1.08; or not Asian: HR = 3.55, and for DFS, HR = 2.87), the data from database (clinical: for OS, HR = 1.24, and for DFS, HR = 1.40; or database: for OS, HR = 2.29, and for DFS, HR = 3.09), paper published year (after 2014: for OS, HR = 1.90;and for DFS,HR = 1.87; or before 2014: for OS, HR = 1.40, and for DFS, HR = 1.22); cancer type (female specific cancer: for OS, HR = 1.41, and for DFS, HR = 0.64; or non-gender specific cancers: for OS, HR = 2.00, and for DFS, HR = 2.95). In addition, according to the database data, we also found that higher E2F1 expression level would lead to worse prognosis of patients, and the results were consistent with the statistical analysis results in the paper.

**Conclusion:**

E2F1 could be served as a prognostic biomarker in cancer patients and higher levels of in cancer patients could predict shorter overall survival and disease-free survival.

**Supplementary Information:**

The online version contains supplementary material available at 10.1186/s12885-023-10865-8.

## Background

E2F family, an important transcription factor family that regulates gene expression, consists of eight members, that can be organized into three subcategories based on function and expression patterns during the cell cycle: activators (E2F1-3), canonical repressors (E2F4-6), and atypical repressors (E2F7 and E2F8) [1]. The E2F family can regulate cell cycle gene expression, inducing cell proliferation [2] of both normal and cancer cell [3], also they have more molecular activities, such as cell differentiation [4], DNA repair, and cell death. E2F1, encoded by the human E2F1 gene that was the first mature transcription activator discovered in the E2F family, is a transcription activator that binds to DNA with dimerization partner (DP) proteins via the E2 recognition site, 5’-TTTC[CG]CGC-3’, found in the promoter region of various genes [5]. The E2F1 domain enables it to act as a transcriptional activator to drive gene expression, determine the orderly transition of cell cycle from G1 phase to S phase, and enhanced cellular proliferation [3], playing a key role in regulating cell proliferation, also it is significant for normal cell homeostasis, uniquely induces apoptosis [6]. E2F1 consisted with CDKs, cyclins, CDK inhibitors and the RB family of proteins forming a cyclin-dependent kinase (CDK)—retinoblastoma (Rb)-E2F1 regulatory axis [2] has been found in almost all cancers. E2F1 was directly implicated with poor prognosis in types of cancer, such as bladder cancer [7], breast cancer [8], ovarian cancer [9], prostate cancer [4], and has been demonstrated to be a key cancer biomarker [5]. Moreover, highly expressed E2F1 was observed in a variety of tumor tissues [9].

Many of the identified genes are well-known E2F family target genes, which are involved in the origin of DNA replication or mitosis [10], resulting in aneuploidy, which is associated with poor prognosis and drug resistance of tumors. The activity of E2F1 has been investigated in connection with RB-dependent control of G1/S progression of the cell cycle. And, components of the RB-E2F pathway have been implicated in promoting aneuploidy in types of cancers, such as breast, bladder, liver, lung and retinal tumors [11]. Therefore, E2F1 expression and/or elevated E2F1 target expression have been linked with poor prognosis [1]. Although there are many articles suggested that the elevate of E2F1 is related to the occurrence of cancer development and prognosis, a few of study for the dysregulation of E2F1 effect on the prognosis of cancer for more effective meta-analysis, which can provide more reliable results compared with a single study and serves as a powerful tool to explain controversial conclusions [12]. For this reason, we evaluated the prognosis value of E2F1 for human cancers in this article based on published data.

## Materials and methods

### Search strategy

In order to obtain all relevant articles, we used the keywords (E2F1) and (‘carcinoma’ OR ‘cancer’ OR ‘tumor’) to search in PubMed, Web of Science, CNKI database and other similar databases by computer for published case–control studies on the prognoses of E2F1 in cancer patients worldwide from the database construction to May, 31^th^, 2022. In addition, we manually searched for related references in some additional papers and reviews. A total of 1282 articles were searched from three databases using keywords. According to the abstract, 45 articles were identified potentially meeting the inclusion criteria. Finally, a total of 17 studies meeting the inclusion criteria were included after reading the full text of 45 articles, and 28 articles with insufficient data and no relation to prognosis were removed.

### Inclusion and exclusion criteria

In order to identify articles suitable for the present study, all enrolled articles should meet the including criteria: (1) case–control study in English only; (2) all patients were gold standard (pathological diagnosis); (3) E2F1 can be derived from tissue, serum, plasma or other human body fluids; (4) complete clinical data and accurate and reliable results; (5) the Hazard Ratio (HR) value and 95% Confidence Interval (CI) of relevant factors are available in the literature HR and 95%CI can be calculated; (6) E2F1 expression and prognostic value for overall survival (OS), relapse-free survival (RFS) or disease-free survival (DFS) were reported.

Additionally, articles that met one of the following terms were removed: (1) repeat publication or no valid data to be extracted or the data is wrong, incomplete or cannot be systematically evaluated; (2) review or experimental animal data; (3) critical articles, conference abstracts or dissertations; (4) the quality of the literature is poor.

### Data extraction and checking

Two researchers (J.L and W.B) independently conducted literature screening and data extraction according to literature inclusion and exclusion criteria, and cross-checked. In case of disagreement, the decision should be made through consultation with each other first. If no agreement can be reached, a third researcher will discuss and make a ruling together. The self-made spreadsheet was used to extract data including first author, publication year, sample size, sample type, etc. Literature quality was evaluated using the Newcastle–Ottawa Scale (NOS), which included eight standard items, including study object selection, inter-group comparability and exposure factor evaluation [13]. The full score was 9, ≥ 7 was high quality literature, 5–6 was medium quality literature, and < 5 was poor quality literature.

During article searching, two essays [14, 15] regarding to lung adenocarcinoma (LAUD) and lung squamous cell carcinoma (LUSC) were from the same database as an included article [15] on lung cancer (LC). In order to prevent repeated inclusion of data, we have deleted the above two essays. We calculated the data from the literature quadratically and included them in our study during the contrary to the conventional method used by S. Mega [16]. OS values were reported in all 17 essays, and DFS [17–21] and RFS [22] were also reported as an indicator of prognosis, which was presented in the figure, but due to only one data, no further analysis was performed.

### Calculated data and subgroup analysis

Among the included 17 articles, there were 12 types of tumors, including women with higher incidence cancer (endometrial cancer, cervical cancer, breast cancer) and non-gender specific cancers (liver cancer, renal cell carcinoma, colorectal cancer, esophageal cancer, esophageal squamous cell carcinoma, cholangiocarcinoma, lung cancer, prostate cancer, high-grade glioma). Therefore, a gender specific cancer subgroup was conducted. In addition, subgroup analysis was conducted by races (Asian or Non-Asian), sample size of patients (≤ 150 or > 150), year of publication (before or after 2014), the data source (clinical or database), survival data (OS or DFS), respectively [23].

### Statistical analyses

The pooled survival of all included patients was calculated with Stata17.0 software. Q test and *I*^2^ test were used to evaluate the statistical heterogeneity of the included essays. If *P* ≥ 0.05 and *I*^2^ ≤ 50% indicated that there was no statistical heterogeneity among essays, fixed-effect model was used for meta-analysis, otherwise, the random effect model was applied. The counting data were described by Hazard ratio (HR) value and 95% Confidence Interval (CI), and the combined HR value was tested by Z test. Sensitive behavior analysis was used for data reliability analysis. To describe the publication bias, funnel plots, Begg’s and Egger’s tests were applied. *P* < 0.05 was considered statistically significant and we marked statistically significant data with *.

### Database analysis

We explored the role of E2F1 in cancer prognosis prediction in the GEPIA (http://gepia.cancer-pku.cn/) and Kaplan–Meier Plotter databases (http://kmplot.com/analysis/), respectively.

## Results

### Eligible studies

**Fig. 1:**
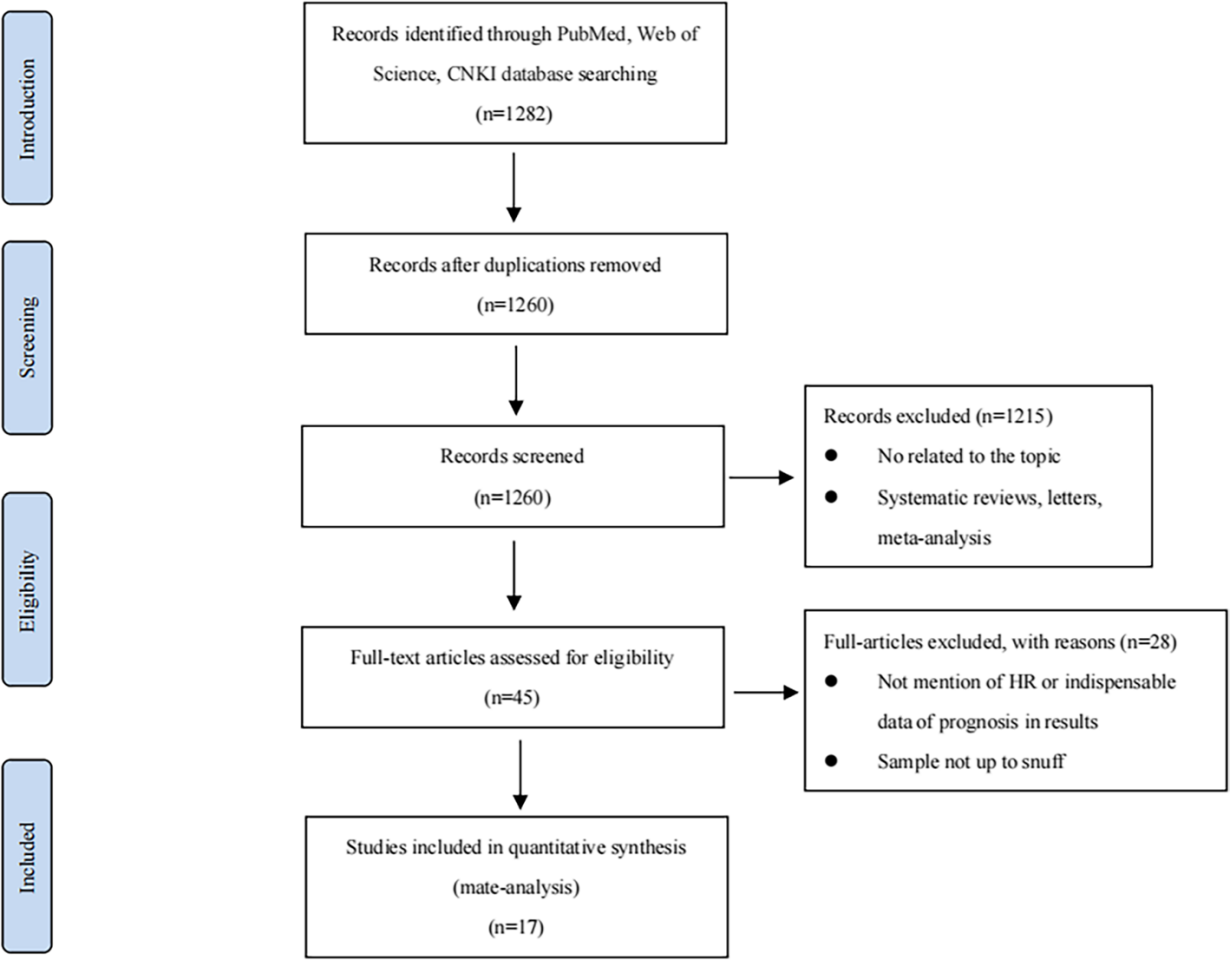
Flow diagram of the study selection process

In this study, a total of 17 articles were enrolled (Supplementary Table 1), eight of which was conducted with clinical samples, and the other nine essays were based on TCGA and other databases. Most of the included articles were published in 2020 or later, so this meta-analysis is time-sensitive (Fig. 1).

### Quality assessment

To assess the quality of included studies, we scored each article strictly according to the scoring standard, and those with a score greater than 6 were considered high-quality articles (Supplementary Table 2). Finally, five articles scored 8, three articles scored 6, and others were scored 7.

### Study characteristics

This study enrolled 17 studies involved in 4481 cases and 725 controls. Among the included articles, 15 studies were conducted based on the population from Asian, two from not Asian, and two had a sample size greater than 150. For the cancer type, a total of 12 tumor types were included in this analysis, four studies concerning three cancers (breast cancer, endometrial carcinoma and cervical carcinoma) with higher incidence rate in women were included in subgroup of female specific cancer, and the remaining 12 studies were grouped in non-gender specific cancer. The clinical samples involved in the article were all tissue samples. In addition, there were three articles published before 2014 and 14 articles published after 2014, respectively. Moreover, eight were from clinical data and 11 were from database data.

### Expression of E2F1 and cancer patients’ survival

**Fig. 2:**
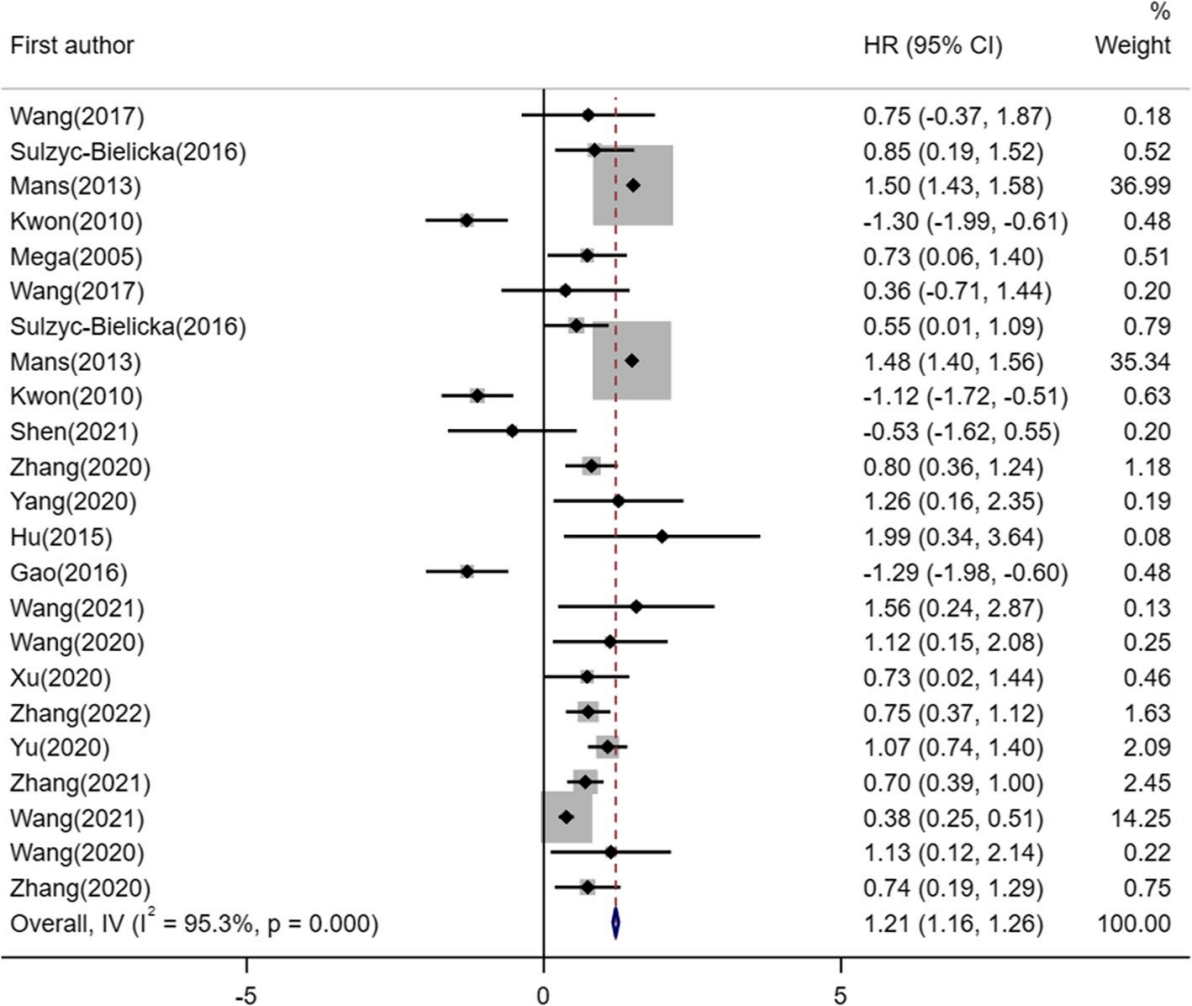
Forest plot of extracted HR for the association of E2F1 expression (*p < 0.05)

The pooled results of all 17 studies conducted with tumor tissue showed that the increased expression of E2F1 was a favorable survival prognosis biomarker (HR = 1.21, 95%CI:1.16–1.26, *I*^2^ = 95.3%, *P_Heterogeneity_ = 0.000) (Fig. 2).

**Fig. 3:**
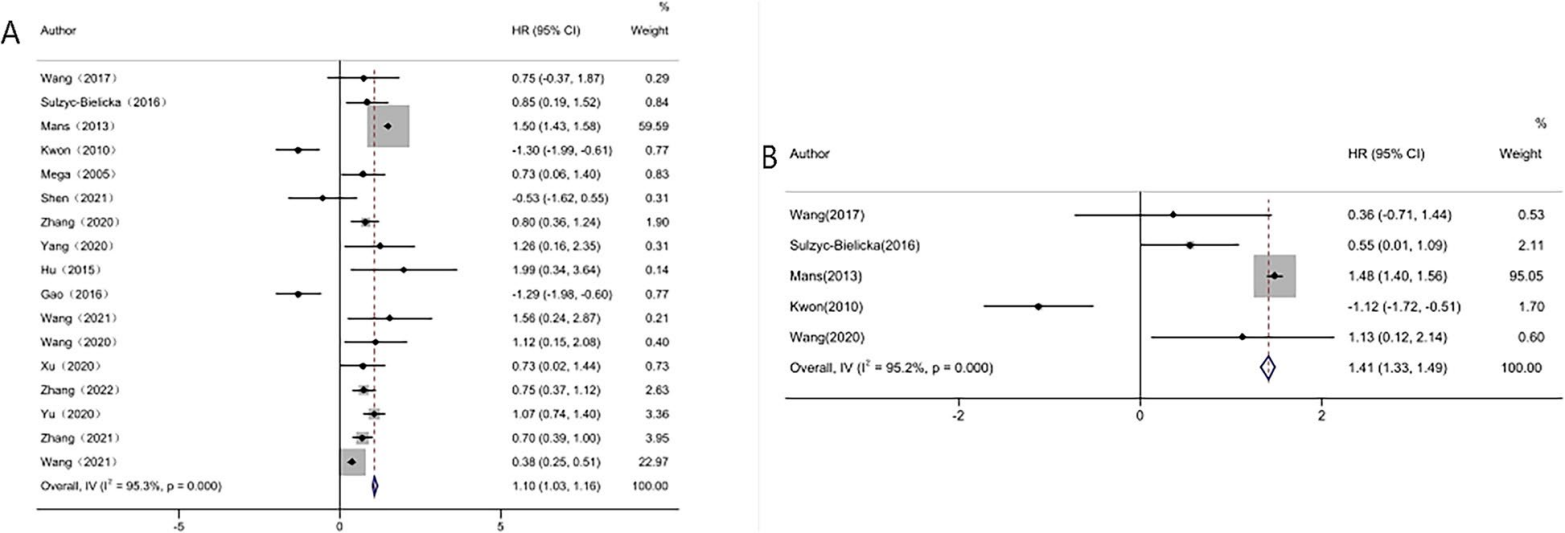
Forest plot of extracted HR for the association of E2F1 expression with OS /DFS. **A** Forest plot of extracted HR for the association of E2F1 expression with OS (*p < 0.05)and (**B**) Forest plot of extracted HR for the association of E2F1 expression with DFS (*p < 0.05)

In this study, a total of 17 studies reported the data of OS, and five studies reported DFS, the pooled results showed that higher E2F1 expression was correlated with unfavorable OS (HR = 1.10, 95%CI:1.03–1.16,* I*^2^ = 95.3%, *P_Heterogeneity_ = 0.000) and DFS (HR = 1.41, 95%CI:1.33–1.49,* I*^2^ = 95.2%, *P_Heterogeneity_ = 0.000) in cancer patients (Fig. 3).

### Subgroup analysis data

According to different criteria, the OS group and DFS group were divided into five subgroups, a significant association was observed in subgroup of number of patients (> 150: HR = 1.77, 95%CI:1.25–2.51), ethnicity (Asian: HR = 1.65, 95%CI:1.19–2.29; not Asian: HR = 3.55, 95%CI:1.92–6.55), the data source (database: HR = 2.29, 95%CI:1.72–3.06); published year (after 2014: HR = 1.90, 95%CI:1.41–2.55) and cancer type (non-gender specific cancers (HR = 2.00, 95%CI:1.30–3.09) for OS. Moreover, in DFS group, a significant association was also observed in subgroup of (< 150: HR = 4.39, 95%CI:4.05–4.76), ethnicity (no Asian: HR = 2.87, 95%CI:1.15–7.14); the data source (database: HR = 3.09, 95%CI:1.12–8.49); published year (after 2014: HR = 1.87, 95%CI:1.21–2.89) and cancer type (non-gender specific cancers: HR = 2.95, 95%CI:1.47–5.91).

### Heterogeneity analysis

**Fig. 4:**
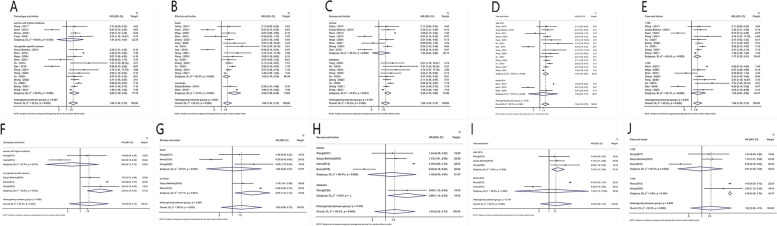
Forest plots of the E2F1 according to subgroup. **A** Cancer type subgroup in OS group (*p* > 0.05). **B** Ethnicity subgroup in OS group (*p < 0.05). **C** Sources subgroup in OS group (*p* > 0.05). **D** Year subgroup in OS group (*p* > 0.05). **E** Case subgroup in OS group (*p* > 0.05). **F** Cancer type subgroup in DFS group (*p* > 0.05). **G** Ethnicity subgroup in DFS group (*p* > 0.05). **H** Sources subgroup in DFS group (*p* > 0.05). **I** Year subgroup in DFS group (*p* > 0.05). **J** Case subgroup in OS group (*p < 0.05)

A significant heterogeneity was observed across enrolled studies. To further explore the source of heterogeneity, we performed a subgroup analysis and the results revealed that, the OS group, the origin of heterogeneity was from ethnicity (*P_Heterogeneity_ = 0.031), but not publication year (*P*_Heterogeneity_ = 0.720), number of patients (*P*_Heterogeneity_ = 0.835), data source (*P*_Heterogeneity_ = 0.165), and tumor type (*P*_Heterogeneity_ = 0.587) (Fig. 4), and that, in DFS group, the origin of heterogeneity was from number of patients (*P_Heterogeneity_ = 0.009), but not publication year (*P*_Heterogeneity_ = 0.747), ethnicity (*P*_Heterogeneity_ = 0.264), data source (*P*_Heterogeneity_ = 0.338), and tumor type (*P*_Heterogeneity_ = 0.062) (Fig. 4).

Within the OS group, there was significant heterogeneity (*P_Heterogeneity_ = 0.031) among ethnicity subgroups. Three articles that may cause great heterogeneity were been deleted respectively and their impact on *P*-value was analyzed, which were 0.066(Gao et al. [24]), 0.066(Kwon et al. [19]) and 0.045(Shen et al. [25]) respectively, indicating A and B contributed the mainly heterogeneity, and after deleting the Gao [24] and Kwon’s [19] articles, the *P* value changed significantly (*P*_Heterogeneity_ > 0.05).

In the DFS group, the *P* value of subgroup based on sample size was less than 0.05 (*P_Heterogeneity_ = 0.009). After deleting Kwon's article [19], heterogeneity within the group was disappear (*I*^2^ = 0.0%, *P*_Heterogeneity_ = 0.766). And, there were significantly association between high expression of E2F1 and the subgroup that non-gender specific cancers (HR = 2.96, 95%CI:1.47–5.91, *I*^2^ = 82.8%, *P_Heterogeneity_ = 0.003), so as the subgroup that the number of patients which less than 150 (HR = 4.39, 95%CI:4.05–4.78, *I*^2^ = 0.0%, *P*_Heterogeneity_ = 0.494).

### Sensitivity analysis

**Fig. 5:**
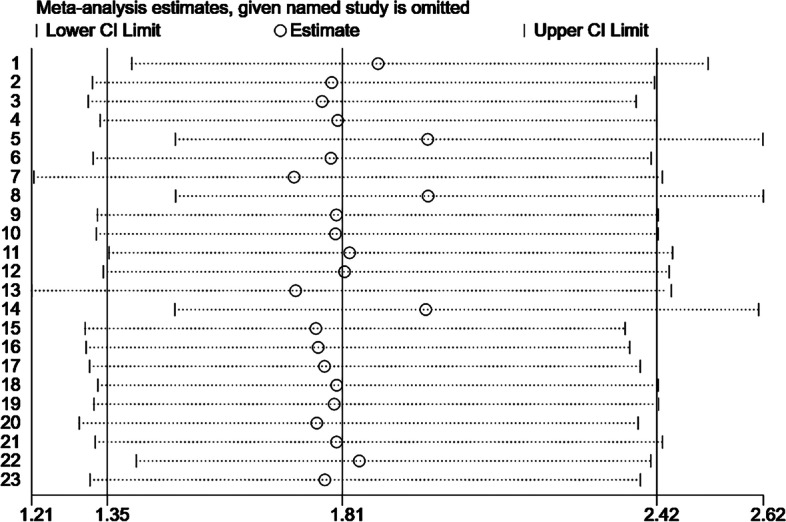
Sensitivity analysis

In order to evaluate the robustness and reliability of the combined results, sensitivity analysis was conducted. One article was successively removed, and the remaining articles were merged by meta-analysis to observe the changes in the combined results and evaluate whether the original meta-analysis results had significantly changed due to the influence of some studies. As shown in the Fig. 5, after most of the essays were excluded, the pooled results of the remaining studies were still statistically significant (95%CI included 1), indicating that the result of this meta-analysis was robustness.

### Publication bias

**Fig. 6:**
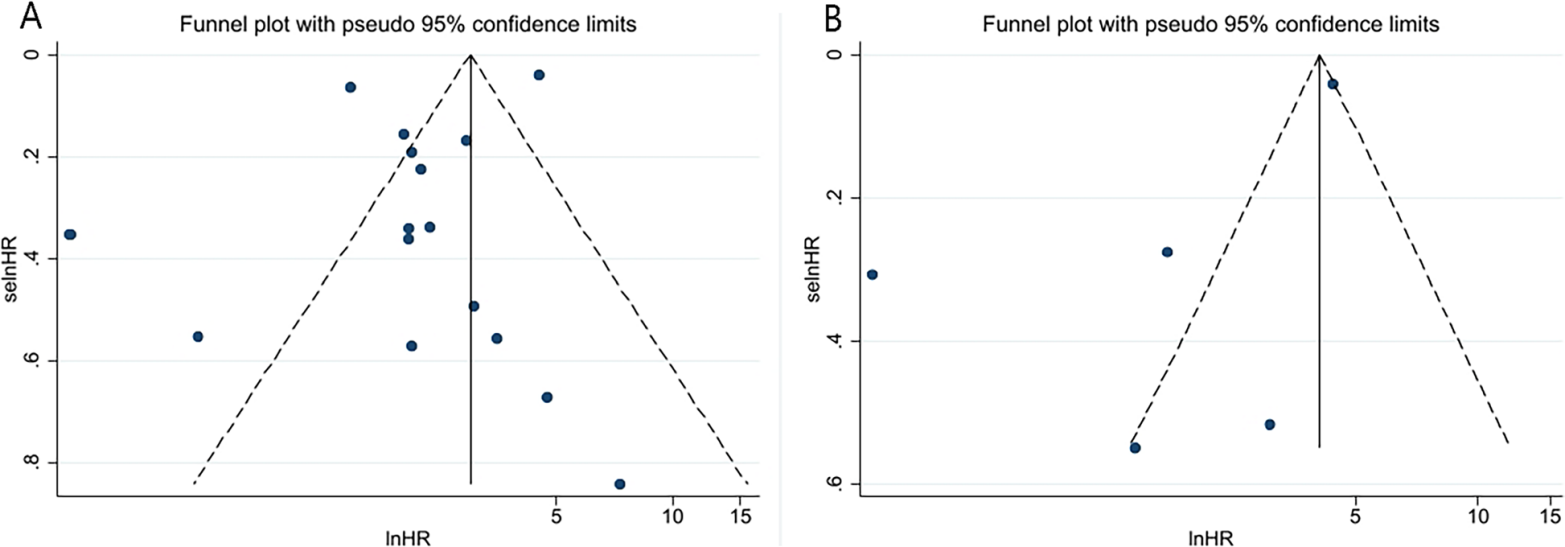
Funnel plot for estimating the publication bias analysis. **A** OS group and (**B**) DFS group

**Fig. 7:**
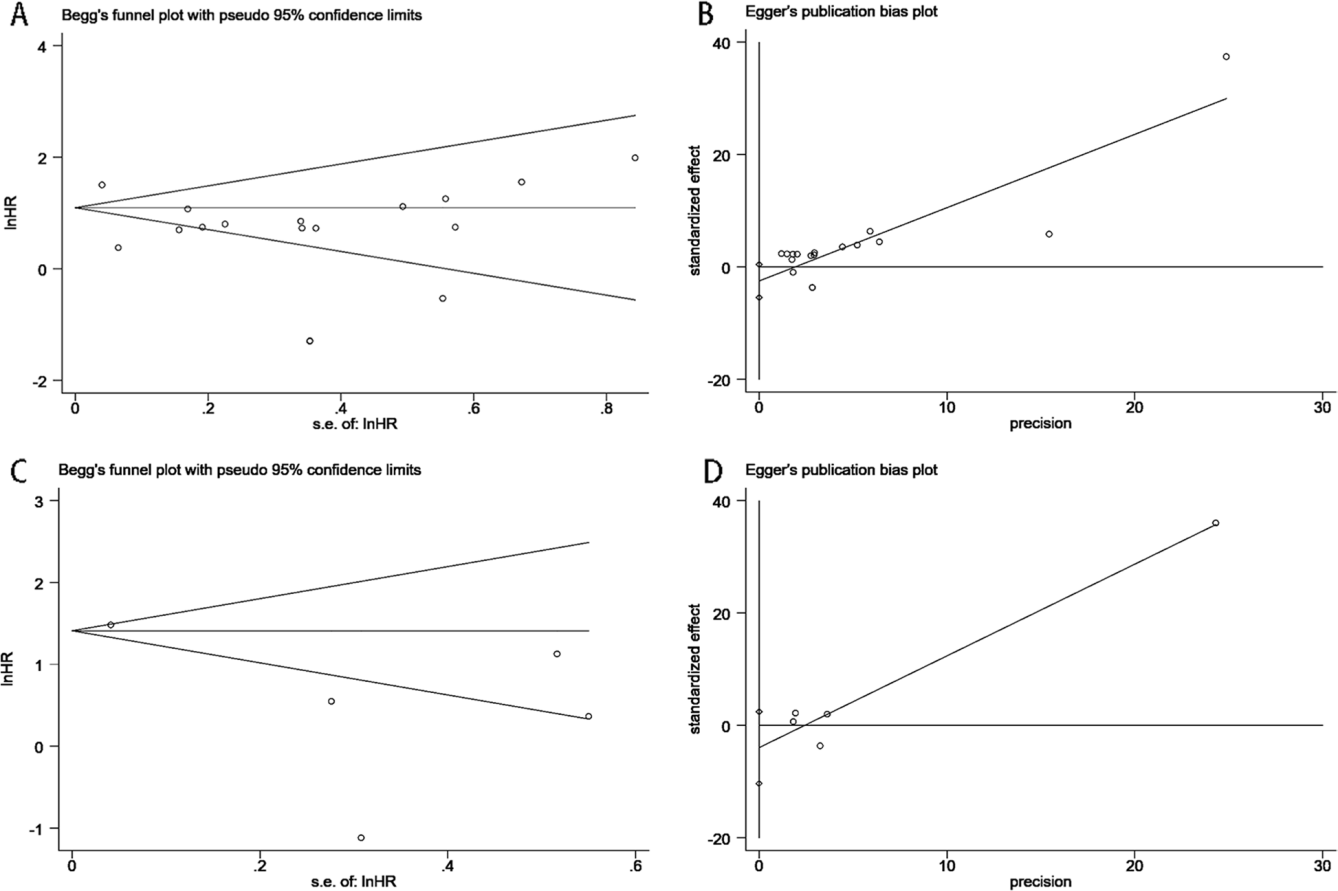
Begg’s tests and Egger’s tests for estimating the publication bias analysis. **A** Begg’s tests for OS group and (**B**) Egger’s test for OS group and (**C**) Begg’s tests for DFS group and (**D**) Egger’s test for DFS group

We conducted funnel plots to detect publication bias in the OS group and the DFS group, respectively, and the results showed that the funnel plots were both asymmetric, indicating possible publication bias, as shown in Fig. 6. In order to fully prove whether publication bias exists in this meta-analysis, Begg’s test and Egger’s test were also conducted in this study, and the results are shown in Fig. 7. The Begg’s test (OS: *P*r >|z|= 0.077; DFS: *P*r >|z|= 0.806) and Egger’s (OS: *P* >|t|= 0.090; DFS: *P* >|t|= 0.142) test results of OS group and DFS group showed that there was no publication bias in either group.

### Prognostic analyses based on the database

**Fig. 8:**
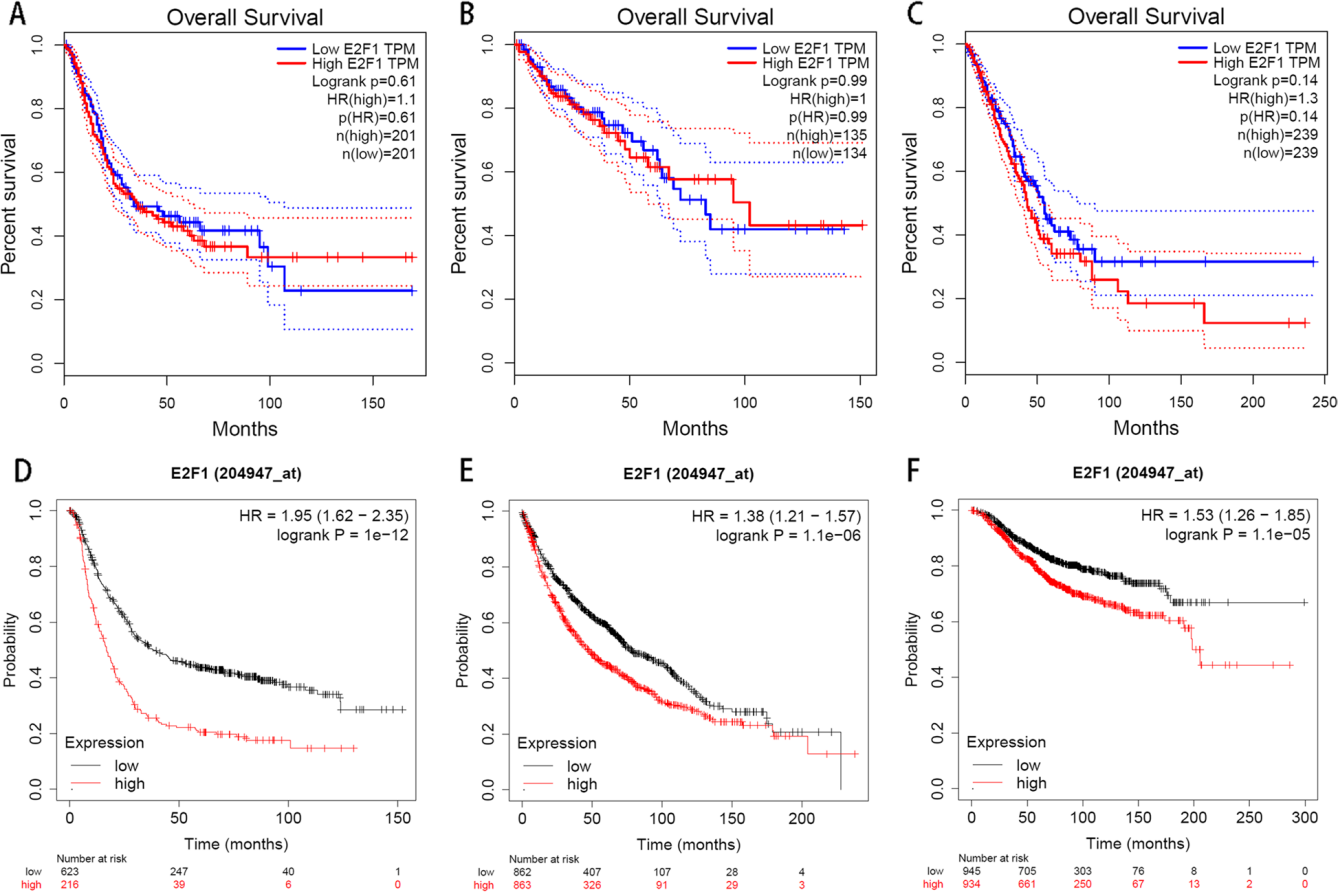
Results from database showed that E2F1 is a valuable prognostic biomarker for cancers Survival prognosis curve (**A**) Bladder Urothelial Carcinoma, (**B**) Colon adenocarcinoma, (**C**) Lung adenocarcinoma; K–M plotter of E2F1 in (**D**) Lung cancer, (**E**) Ovarian cancer, (**F**) Breast cancer

In order to verify the prognosis of E2F1 for cancer, we searched in the online databases GEPIA, which contains survival and differential expression analyses of genes and in Kaplan–Meier Plotter database, which includes the effect of genes, protein on survival in 21 cancer types. As shown in Fig. 8, the prognostic HR values of E2F1 in patients with Bladder Urothelial Carcinoma, Colon adenocarcinoma, and Lung adenocarcinoma were 1.10 (*P* = 0.61), 1.00 (*P* = 0.99), 1.3 (*P* = 0.14), respectively. In addition, the results of Kaplan–Meier Plotter database showed that high expression of E2F1 predicted unfavorable OS of lung cancer patients, ovarian cancer patients, and breast cancer patients (HR = 1.95, 95% CI: 1.62–2.35, *P* = 1e-12; HR = 1.38, 95% CI:1.21–1.57, *P* = 1.1e-06; HR = 1.53, 95% CI: 1.26–1.85, *P* = 1.1e-05). Therefore, all the results from databases supported the pooled results based on published data.

## Discussion

In this study, we evaluated the prognostic value of E2F1 in cancer patients according to published data. The results showed that higher E2F1 level was significantly correlated with unfavorable OS and DFS in cancer patients.

In fact, previous studies have found that E2F1 expression was elevated in tumor tissues and associated with the poor prognosis of cancer. The expression levels of E2Fs (E2F1-8) were all significantly up-regulated in LUAD tissues [14], Wang et al. investigated the expression, prognosis value, mutation, and potential relationship with immunological aspects of E2Fs in LUAD by bio-informatics analysis [14].E2F1 is expressed strongly in 59.8% of ESCC and that the over-expression was correlated with tumor progression, lymph node metastasis, and poor prognosis after surgery [16]. Over-expression of E2F1 was significantly associated to worsen OS in all NSCLC patients followed for 200 months, as well as in LUAD patients [26],which was duplicated in HCC patients [27].

The pooled results indicated that there was a significant association between elevated E2F1 and cancer patients’ poor survival (OS/DFS). However, there was significant heterogeneity among the enrolled studies. The two articles conducted by Kwon [19] and Gao [24] were the origin of heterogeneity for subgroup of the OS group, which was attributed to: (1) the types of patients included were different in that the patients included in Kwon's [19] paper were patients with surgically treated, while the rest of the literature included patients with untreated; (2) tumor size varies: most of the patients with tumor size > 4 were included in Kwon et al. [19] tumor size > 4 cm; (3) the difference of statistical methods: for example, statistical data reported by Kwon et al. [19] was analysis with Pearson’s v2 test, indicating that the clinical state of patient, pathology of tumor, and statistical analysis methods may affect the result.

The number of patients in the DFS group maybe the origin of heterogeneous which could be eliminated by removing one study coming from Kwon [19]. The possible reason is: (1) the age ratio of the included patients varied: patients older than 60 years in the article reported by Mans et al. [20], whereas, most of the patients included in the other articles [17–19, 21] were younger than 60 years old; (2) different stages of tumor development occur: the included tumor samples were mostly infiltrated by Wang et al. [17] and had distant metastasis, while the tumor samples in other literature were mostly in the early or middle stages. Therefore, more attention should be paid on the age of the included patients and the status of the included sample.

E2F1 is a prognostic marker in a variety of cancers, because E2F1 is involved in multiple regulatory pathways, which also the reason for its elevated expression in the tumor micro-environment. A large number of essays have investigated the involvement of E2F1 in protein regulation and thus the impact of tumor development and prognosis. The distinct regulatory networks involving E2F1 in HCC with different backgrounds may contribute to the modulation of expression profiles due to different etiological factors [28]. E2F1 transactivates IQGAP3, and IQGAP3 competitively inhibits the interaction between PKCδ and PKCα, resulting in phosphorylation and activation of PKCα and promotion of cell proliferation in HCC cells [29]. The YY1/ miR-526b-3p/E2F1 axis as a pathway for abnormal E2F1 expression in CRC [30]. An over-activated NFYB-E2F1-CHK1 axis deteriorates the therapeutic effects of oxaliplatin, while the side-effect of chemotherapy persists, likely leading to a worse prognosis [31].

This analysis has several strengths. First, the analysis increases the strength of the current evidence by providing a large sample. Second, the included studies are widely representative, with data from five different countries. In addition, sensitivity analysis and publication bias suggest that the results of this study are robust. However, the study has some limitations. First of all, only summarized data rather than individual patient data were pooled in our study, which might preclude us from conducting a more in-depth analysis; most of the included studies were retrospective studies, and more prospective studies are still needed to validate our analysis. Second, some studies with negative results may not be published, which may lead to potential publication bias. Data from some studies did not specify the included patient information and tumor stage. Therefore, more detailed study designs and follow-up are still needed to explore the prognostic value of E2F1 in cancer patients.

## Conclusion

In short, our study concluded that E2F1 can be used as a prognostic marker for cancers.

## Supplementary Information

**Additional file 1: Supplementary Table 1.** The main features of 17 included studies in prognostic meta-analysis. **Supplementary Table 2.** New castle–Ottawa quality assessments scale.

## Data Availability

The data are available from the corresponding author (B.H) upon reasonable request. All data generated or analyzed during the present study are included in this published article.

## Abbreviations

CI: Confidence interval
OS: Overall Survival
HR: Hazard Ratio
RFS: Recurrence/relapse-free survival
DFS: Disease-free interval
UL: Upper Limit
LL: Lower Limit
NOS: Newcastle–Ottawa quality assessments scale
GC: Gastric cancer
LAUD: Lung adenocarcinoma
LUSC: Lung squamous cell carcinoma
GEO: Gene Expression Omnibus
HCC: Hepatocellular carcinoma
HR: Hazard ratio
NSCLC: Non-small cell lung cancer
TCGA: The cancer genome atlas
