# Supplementary material for: Prognostic role of E2F1 gene expression in human cancer: a meta-analysis
Source: BMC Cancer. 2023 Jun 5;23:509. doi: 10.1186/s12885-023-10865-8 (PMC10243032; doi:10.1186/s12885-023-10865-8)
Supplement: Supplementary file 1 — Additional file 1: Supplementary Table 1. The main features of 17 included studies in prognostic meta-analysis. Supplementary Table 2. New castle–Ottawa quality assessments scale. [file 12885_2023_10865_MOESM1_ESM.docx]

Supplementary Table1 **The main features of 17 included studies in prognostic meta-analysis**

| **First author** | **Year** | **Country** | **Ethnicity** | **Cancer type** | **Case** | **Outcome** | **HR** | **UL** | **LL** | ***P*-value** |
| --- | --- | --- | --- | --- | --- | --- | --- | --- | --- | --- |
| Wang^[17a](#_ENREF_18)^ | 2017 | China | Asian | Cervical cancer | 184 | OS | 2.11 | 6.5 | 0.69 | >0.05 |
| Bielicka^[17b](#_ENREF_19)^ | 2016 | Poland | European | Colorectal cancer | 190 | OS | 2.35 | 4.58 | 1.21 | 0.01 |
| Mans^[17d](#_ENREF_21)^ | 2013 | USA | American | Renal cell carcinoma | 138 | OS | 4.5 | 4.8 | 4.1 | 0.01 |
| Kwon^[17c](#_ENREF_20)^ | 2010 | South Korea | Asian | Breast cancer | 183 | OS | 0.273 | 0.547 | 0.137 | 0.001 |
| Mega^[16](#_ENREF_17)^ | 2005 | Japan | Asian | Esophageal squamous cell carcinoma | 122 | OS | 2.074 | 4.049 | 1.062 | 0.0326 |
| Shen^[19](#_ENREF_24)^ | 2021 | China | Asian | Renal cell carcinoma | 97 | OS | 0.588 | 1.741 | 0.199 | 0.313 |
| Zhang^[18](#_ENREF_23)^ | 2020 | China | Asian | Endometrial cancer | 27 | OS | 2.23 | 3.46 | 1.43 | 0.00026 |
| Yang^[20](#_ENREF_25)^ | 2020 | China | Asian | Cervical cancer | 88 | OS | 3.51 | 10.469 | 1.177 | 0.001 |
| Hu^[21](#_ENREF_26)^ | 2015 | China | Asian | Renal cell carcinoma | 65 | OS | 7.311 | 38.16 | 1.401 | 0.004 |
| Gao^[22](#_ENREF_27)^ | 2016 | China | Asian | Renal cell carcinoma | 114 | OS | 0.2751 | 0.5498 | 0.1376 | 0.0003 |
| Wang^[23](#_ENREF_28)^ | 2021 | China | Asian | Prostate cancer | 499 | OS | 4.74 | 17.7 | 1.27 | 0.013 |
| Wang^[17e](#_ENREF_22)^ | 2020 | China | Asian | Cholangiocarcinoma | 65 | OS | 3.052 | 8.023 | 1.161 | 0.024 |
| Xu^[24](#_ENREF_29)^ | 2020 | China | Asian | Esophageal cancer | 179 | OS | 2.07 | 4.22 | 1.02 | 0.04 |
| Zhang^[25](#_ENREF_30)^ | 2022 | China | Asian | Hepatocellular carcinoma | 208 | OS | 2.11 | 3.07 | 1.45 | 0.001 |
| Yu^[26](#_ENREF_31)^ | 2020 | China | Asian | High-grade glioma | 388 | OS | 2.924 | 4.074 | 2.098 | 0.001 |
| Zhang^[27](#_ENREF_32)^ | 2021 | China | Asian | Renal cell carcinoma | 530 | OS | 2.01 | 2.73 | 1.48 | 4.6E-06 |
| Wang^[15](#_ENREF_16)^ | 2021 | China | Asian | Lung cancer | 486 | OS | 1.46 | 1.65 | 1.28 | 5.9E-09 |

OS: Overall Survival; HR: Hazard Ratio; UL: Upper Limit; LL: Lower Limit

Supplementary Table 2 **Newcastle–Ottawa quality assessments scale**

| **First author** | **Year** | **Quality indicators from NOS** | | | | | | | | **Scores** |
| --- | --- | --- | --- | --- | --- | --- | --- | --- | --- | --- |
|  |  | **1** | **2** | **3** | **4** | **5** | **6** | **7** | **8** |  |
| Shen | 2021 | + | + | + | - | + | - | + | + | 6 |
| Zhang | 2020 | + | + | + | - | ++ | + | + | + | 8 |
| Yang | 2020 | + | + | + | - | ++ | - | + | + | 7 |
| Wang | 2017 | + | + | + | - | ++ | + | + | + | 8 |
| Gao | 2016 | + | + | + | - | + | - | + | + | 6 |
| Bielicka | 2016 | + | + | + | - | ++ | - | + | + | 7 |
| Mans | 2013 | + | + | + | - | ++ | + | + | + | 8 |
| Kwon | 2010 | + | + | + | - | ++ | + | + | + | 8 |
| Mega | 2005 | + | + | + | - | + | + | + | + | 7 |
| Hu | 2015 | + | + | - | - | ++ | + | + | + | 6 |
| Wang | 2021 | + | + | - | - | ++ | + | + | + | 7 |
| Wang | 2020 | + | + | - | - | ++ | + | + | + | 7 |
| Xu | 2020 | + | + | - | - | ++ | + | + | + | 7 |
| Zhang | 2022 | + | + | - | - | ++ | + | + | + | 7 |
| Yu | 2020 | + | + | - | - | ++ | + | + | + | 7 |
| Zhang | 2021 | + | + | - | - | ++ | + | + | + | 7 |
| Wang | 2021 | + | + | - | - | ++ | + | + | + | 7 |

1. Representativeness of the exposed cohort; 2. Selection of the non-exposed cohort; 3. Ascertainment of exposure; 4. Outcome of interest not present at the start of study; 5. Control for important factor or additional factor; 6. Assessment of outcome; 7. Follow-up long enough for outcomes to occur; 8. Adequacy of follow-up of cohorts.
